# Genome-Wide Assessment Characteristics of Genes Overlapping Copy Number Variation Regions in Duroc Purebred Population

**DOI:** 10.3389/fgene.2021.753748

**Published:** 2021-10-14

**Authors:** Zhipeng Wang, Yuanyuan Guo, Shengwei Liu, Qingli Meng

**Affiliations:** ^1^ College of Animal Science and Technology, Northeast Agricultural University, Harbin, China; ^2^ Bioinformatics Center, Northeast Agricultural University, Harbin, China; ^3^ Beijing Breeding Swine Center, Beijing, China

**Keywords:** copy number variations, duroc purebred pig, CNV-miRNAs, CNV-genes, genome-wide assessment

## Abstract

Copy number variations (CNVs) are important structural variations that can cause significant phenotypic diversity. Reliable CNVs mapping can be achieved by identification of CNVs from different genetic backgrounds. Investigations on the characteristics of overlapping between CNV regions (CNVRs) and protein-coding genes (CNV genes) or miRNAs (CNV-miRNAs) can reveal the potential mechanisms of their regulation. In this study, we used 50 K SNP arrays to detect CNVs in Duroc purebred pig. A total number of 211 CNVRs were detected with a total length of 118.48 Mb, accounting for 5.23% of the autosomal genome sequence. Of these CNVRs, 32 were gains, 175 losses, and four contained both types (loss and gain within the same region). The CNVRs we detected were non-randomly distributed in the swine genome and were significantly enriched in the segmental duplication and gene density region. Additionally, these CNVRs were overlapping with 1,096 protein-coding genes (CNV-genes), and 39 miRNAs (CNV-miRNAs), respectively. The CNV-genes were enriched in terms of dosage-sensitive gene list. The expression of the CNV genes was significantly higher than that of the non-CNV genes in the adult Duroc prostate. Of all detected CNV genes, 22.99% genes were tissue-specific (TSI > 0.9). Strong negative selection had been underway in the CNV-genes as the ones that were located entirely within the loss CNVRs appeared to be evolving rapidly as determined by the median dN plus dS values. Non-CNV genes tended to be miRNA target than CNV-genes. Furthermore, CNV-miRNAs tended to target more genes compared to non-CNV-miRNAs, and a combination of two CNV-miRNAs preferentially synergistically regulated the same target genes. We also focused our efforts on examining CNV genes and CNV-miRNAs functions, which were also involved in the lipid metabolism, including *DGAT1, DGAT2, MOGAT2*, miR143, miR335, and miRLET7. Further molecular experiments and independent large studies are needed to confirm our findings.

## Introduction

Recent findings have shown that structural DNA variations are widespread in animal genomes, such as those of rodents (Graubert et al., 2007) and primates (Freeman et al., 2006). The copy number variation (CNV) has been considered a major type of structural variations, with a length ranging from one to several Mb (Feuk et al., 2006). With recent advances in high-throughput sequencing technologies, various approaches can be applied to perform genome-wide CNV mapping, including DNA hybridization in BAC/PAC/oligonucleotide arrays, SNP chips, and next-generation sequencing. Using genome-wide technologies of higher resolution, tremendous quantities of CNVs have been identified in many farm animal species, such as cattle (Liu et al., 2010; Mei et al., 2020), pig (Ramayo-Caldas et al., 2010; Jiang et al., 2014; Wang et al., 2015a), sheep (Liu et al., 2013; Zhu et al., 2016; Di Gerlando et al., 2019), and chicken (Griffin et al., 2008; Seol et al., 2019).

As in other domestic animals, reliable detection of CNVs in swine is still challenging, with a low concordance among different studies. Some evidence suggests that multiple populations should be surveyed to construct an accurate CNV map (Liu et al., 2010; Ramayo-Caldas et al., 2010). Identifying CNVs from different genetic backgrounds can validate the data on CNV regions discovered in various investigations and achieve reliable CNVs mapping that describes the genome-wide characteristics of various populations.

By molecular mechanisms, such as gene disruption, gene fusion, positive effect, and dosage effect, CNVs can cause Mendelian disease or traits, or be associated with complex disease or quantitative traits (Lupski and Stankiewicz, 2005). CNVs affect the phenotypic variation in domestic animal genomes. For example, (Fliskowski et al., 2010) identified a 110 kb deletion of the *MIMT1* gene in the cattle genome, which was associated with abortions and stillbirth phenotype. The dominant white color of swine has been associated with a duplication of a 450 kb fragment encompassing the *KIT* gene (Giuffra et al., 2002; Seo et al., 2007). Recent studies found a high frequency in miRNA copy number abnormality. In this respect, (Marcinkowska et al., 2011) detected miRNAs located in the human CNVR that also had potential functional variants. Moreover, (Willemsen et al., 2011) reported a deletion of 1p21.3 containing MIR137, which induced miRNA downregulation and upregulation of targets in subjects with congenital abnormalities. The aforementioned examples reveal the association between copy number change and gene function, which leads to alteration of some phenotypes. Thus, characteristics of genes overlapping CNVRs are to be investigated, and the potential regulatory mechanisms of these genes are to be analyzed and established.

In this study, we performed genome-wide CNVR mapping in a Duroc swine population using a 50 K SNP Chip. Our findings provide a useful complement swine genomic structure variations and validate CNVs detected in previous investigations. Furthermore, we have presented the structure and characteristics of protein-coding genes (CNV genes) or miRNAs (CNV-miRNAs) overlapping the CNV map and had discussed in detail the impact of CNVRs on gene morphology and function.

## Materials and Methods

### Animal Population

A total number of 208 Duroc pigs (10 males and 198 females) were used in this study, which were obtained from the whole foundation herd of the Beijing Breeding Swine Center. The pigs were located on the same farm, under similar environmental conditions and an identical standard feeding schedule. All animals were inspected for the presence of open wounds, any illness, or abnormal behavior. All pigs are alive and without genetic modification.

### Single Nucleotide Polymorphism (SNP) Genotyping and Quality Control

We used the phenol-chloroform method to extract genomic DNA from blood. Genotyping of a total number of 50,703 SNPs across the whole genome was performed using the GeneSeek Porcine 50 K SNP Chip (Neogen, Lincoln, NE, United States ). We performed the following quality control through PLINK (V1.90) software (Purcell et al., 2007) and determined the numbers of SNPs in the following categories: 1) SNPs with minor allele frequencies (MAF) ≥5% and 2) SNPs and individual call rates ≥95%. Only autosomal SNPs, with a total number of 40,070 SNPs, were considered for subsequent analyses. The genotyping module of BeadStudio tool (Illumina, Inc., San Diego, CA, United States) was used to determine the genotypes signal intensity of the individuals, including log R ratio (LRR) and B-allele frequency (BAF).

### Identification of Swine CNVs and CNVRs

In the present study, the PennCNV (Wang et al., 2007) algorithm was used to identify porcine CNVs. Based on the hidden Markov model (HMM), this algorithm can detect CNVs from SNPs genotyping data, which includes abundant information including the signal intensity and the population frequency (PFB) at each SNP marker, and the distance between SNPs, based on the *Sus scrofa* (Sscrofa11.1) genome assembly. To salvage the sample affected by genomic wave, a porcine GC-model file was created by calculating the GC content of the 1 Mb region surrounding each SNP and the -gcmodel option in PennCNV was used for adjustment. After detection of CNVs, PennCNV quality filters were used with the following cutoff values: 1) Standard deviation of LRR < 0.30; 2) BAF drift < 0.01; and 3) Waviness factor value within ± 0.05. To reduce the false positive rate, we acquired a CNV containing three or more consecutive SNPs. Referring to the criteria of (Redon et al., 2006), CNV regions (CNVRs) were determined by aggregating overlapping CNVs identified across all samples, which had to be present in at least two individuals. We divided the CNVRs into three types, including gains, losses, and both types (including gain and loss events).

To verify the CNVs identified by PennCNV, we used the QuantiSNP software (Colella et al., 2007) to analyze the same data set as well. The QuantiSNP algorithm assumes an Objective Bayes hidden-Markov model to improve the accuracy of CNVs identification and mapping, and uses a fixed rate of heterozygosity for every SNP. This CNV calling software was run under default parameters. All CNV calls with a Log Bayes Factor <10 were removed.

Using regression analysis, we assessed the relationship between the numbers of CNVRs and the length of each chromosome. From the results of (Feng et al., 2017), we obtained the segmental duplication (SD) regions of the swine genome, and analyzed the relationship between CNVRs and SD using Chi-squared test. At the same time, we used Chi-squared test to compare gene density between CNV regions and non-CNV regions.

To date, only 20 studies have been focused on genome-wide CNV identification in pigs. Of them, two studies employed Sscrofa9.2, and 17 utilized Sscrofa10.2 genome, respectively. To increase the accuracy of the comparisons among studies, CNVRs located on the Sscrofa9.2 and Sscrofa10.2 assembly were converted into the Sscrofa11.1 genome using NCBI Remap tools.

### Function Annotation and Analysis of CNVRs

Swine transcripts and annotations were downloaded from the Ensembl database. According to the position of the CNVRs and genes, we identified the protein-coding genes and miRNA partially or completely overlapping with the CNVRs. The DAVID Bioinformatics Resources was used for function analysis, including Gene Ontology (GO) and Kyoto Encyclopedia of Genes and Genomes (KEGG).

Based on the structural relationships between protein-coding genes and CNVRs, we classified the genes into three types, as previously suggested by Woodwark and Bateman (2011). Type I CNV-gene was contained entirely within the CNV. Type II CNV-gene partially overlapped the CNV, which were often disrupted and even with fusion genes formed. Type III genes were those that contained the CNV within the gene. To better understand the biology of the aforementioned three types of CNV genes, we investigated their basic characteristics, selective pressures, and functional annotation. The dN and dS values of the pig/human ortholog were obtained from Ensembl Compare database using PAML. We used the Kolmogorov-Smirnov test to compare dN or dS value among three type genes.

Next, we curated dosage-sensitivity gene list, including the imprinted genes, monoallelically genes. These genes were taken from the Database of Chromosomal Imbalance and Phenotype in Humans using Ensembl Resources (DECIPHER, http://decipher.sanger.ac.uk/index) (Firth et al., 2009), the International Standards for Cytogenomic Arrays (ISCA, http://www.iscaconsortium.org) (Riggs et al., 2012), the Catalogue of Parent of Origin Effects database (Morison et al., 2001; Gimelbrant et al., 2007), and the Geneimprint database (www.geneimprint.com) (Chen and Begcy, 2020). The swine genome contains 21 and 369 imprinted and monoallelically expression genes, correspondingly. Based on data from the Ensembl Genome Compare database, we selected the porcine ortholog gene with human dosage-sensitivity genes. Overall, we established a total number of 1,542 dosage-sensitive genes in swine genome, including 166 imprinted genes, 1,043 monoallelically expressed genes.

The sequenced RNA-seq raw data of 27 adult Duroc tissue types, including retina, pancreas, gut, brain, gall bladder, lung, liver, testes, salivary gland, longissimus dorsi, spinal cord, thyroid, lymph, urinary bladder, spleen, prostate, kidney, adrenal gland, esophagus, stomach, heart, nasopharynx, fat, ovary, breast, placenta, and uterus, were downloaded from NCBI SRA (Sequence Read Archive) database with the BioProject number PRJNA392949 (Zhao et al., 2018). After the QC step conducted using FASTQC, Trimmomatic tools (v3.6), RNA-seq clean data were mapped to the *Sus scrofa* 11.1 genome release version with Hisat2. To obtain expression levels of all genes in the samples of each of the tissue types, fragments per kilobase of exon model per million mapped reads (FPKM) and counts were calculated using StringTie 1.3.4 and FeatureCounts1.6.0 tools, respectively. We analyzed the difference expression profiles between CNV and non-CNV genes in each tissue. To decrease false positive, we adjusted *p*-value using the Bonferroni method, which the threshold is 1.85E-3.

We used the tissue specificity index (*τ*) (Yanai et al., 2005) to grade the scalar measurements of the expression specificity, which ranged from 0 for housekeeping genes to 1 for tissue-specific genes. The index *τ* is defined as $\text{τ}=\frac{\Sigma_{i=1}^{N}\left(1-{\text{x}}_{\text{i}}\right)}{\text{N}-1}\;$ , where N is the number of tissues, and x_i_ is the expression normalized by the maximal expression value.

We used the miRanda tool (Betel et al., 2008) to predict the target gene regulated by miRNAs. The miRanda algorithm integrated biological knowledge on target rules of mammalian microRNAs. In this study, Tot Score and Tot Energy values set 140 and −20, respectively.

To identify the target-recognition preference of miRNAs overlapped CNVR, we employed a random sampling method, based on the procedure proposed by (Wu et al., 2012). The simulation process included two steps: 1) CNV-miRNAs were randomly selected from all miRNAs in the porcine genome, called pseudo-CNV-miRNAs; 2) Based on the relationship between miRNA and the target genes predicted by miRanda, we marked the relationships target genes and pseudo-CNV-miRNAs or pseudo-non-CNV miRNAs, respectively; 3) For each regulatory type, we re-recorded the number of target genes. Steps (a)–(c) were repeated 10,000 times.

In this study, all statistical analyses, including regression analysis, Kolmogorov-Smirnov test, Wilcoxon rank-sum test, Fisher’s exact test, and Chi-squared test were performed using R4.0.0 software.

## Results

### Identification and Characterization of CNVs on Duroc Genome

**FIGURE 1:**
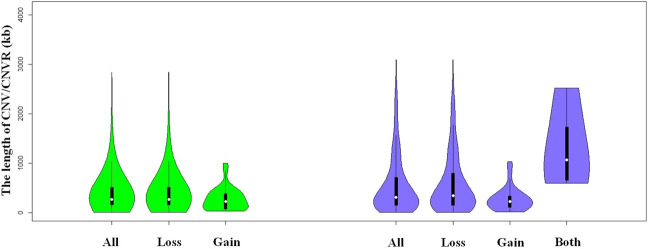
Violin plots of the total CNVs (CNVRs) lengths, gain or loss CNVs (CNVRs) lengths, and both CNVRs lengths in Duroc purebred population.

We identified a total number of 1,371 CNVs within the autosome genome of Duroc populations (Table 1), whose sizes ranged from 8.37 to 2,838.50 kb. The average and the median sizes were 386.30 and 270.05 kb, respectively. The copy number losses were 28.17 times more frequent than the copy number gains (1,324 losses versus 47 gains). The size of the CN losses and CN gains ranged from 8.37 to 2,838.50 kb and from 33.37 to 998.00 kb, respectively. The median and average sizes of the CN losses (270.05 and 390.39 kb) were slightly larger than those of the CN gains (223.71 and 271.03 kb). The distribution of CNVs size ranges are illustrated in Figure 1. In this Duroc swine population, 205 individuals had CNVs, whereas three individuals were without CNVs, with an average number of CNVs per individual genome of 6.59.

**TABLE 1:** CNVR distributions in the each chromosome of Duroc purebred population.

SSC	Length (Mb)	CNV			CNVR			
		Total	Gain	Loss	Total	Gain	Loss	Both
1	274.33	74	0	74	14	0	14	0
2	151.94	108	4	104	17	4	13	0
3	132.85	190	4	186	9	2	6	1
4	130.91	81	2	79	8	1	7	0
5	104.53	49	0	49	13	0	13	0
6	170.84	179	1	178	14	1	13	0
7	121.84	75	2	73	15	1	13	1
8	138.97	86	1	85	11	1	10	0
9	139.51	24	5	19	14	4	9	1
10	69.36	27	1	26	6	1	5	0
11	79.17	28	13	15	14	5	9	0
12	61.6	82	0	82	9	0	9	0
13	208.34	117	1	116	9	0	9	0
14	141.76	95	1	94	13	1	12	0
15	140.41	75	3	72	18	2	15	1
16	79.94	40	5	35	12	5	7	0
17	63.49	29	2	27	8	2	6	0
18	55.98	12	2	10	7	2	5	0
Total	2,265.77	1,371	47	1,324	211	32	175	4
Average^a^	—	6.59	0.23	6.37	1.01	0.15	0.84	0.02

a At sample level, each sample has 6.59 (1,371/208) CNVs for Duroc.

### Characteristics of Porcine CNVRs on Duroc Genome

By aggregating the overlapping CNVs, a total number of 211 CNVRs across the autosomes were identified, which covered 118.48 Mb of the swine genome and corresponded to 5.23% of the length of the autosomal sequence. Of these CNVRs, 175 were losses and 32 gains, whereas four contained both events which were within the following ranges 8.34–3,882.48 kb, 19.98–1,035.01 kb, and 596.40–2,516.40 kb, respectively. The CNVRs of losses, gains, and both events had means or medians of 596.30, 277.46, and 1,312.96 or 1,069.49 kb, correspondingly. The distribution of the CNVRs size ranges is depicted in Figure 1. In this study, the loss events were approximately 5.47-fold more common than the gain events.

**FIGURE 2:**
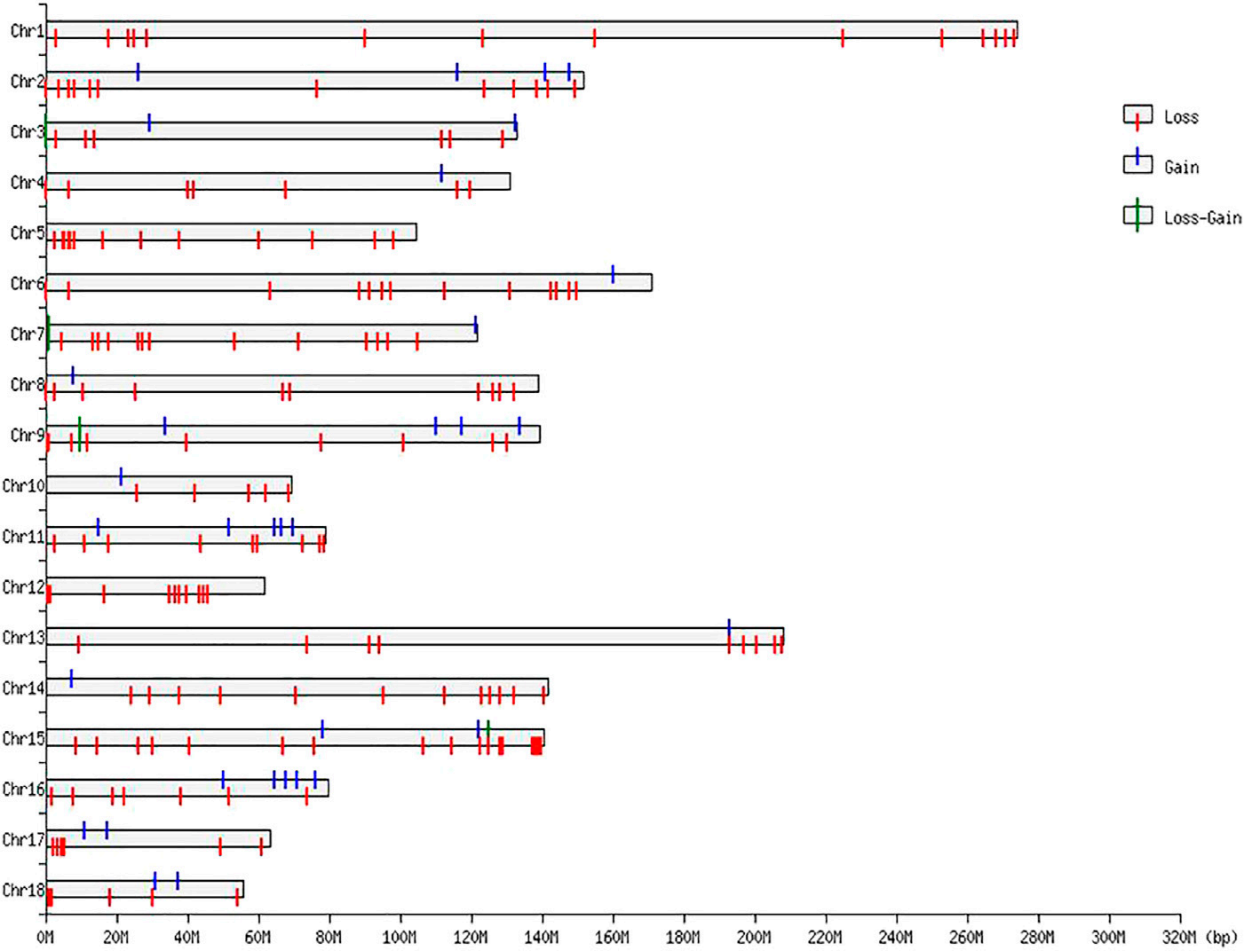
CNVR distributions in the genome of Duroc purebred population.

**FIGURE 3:**
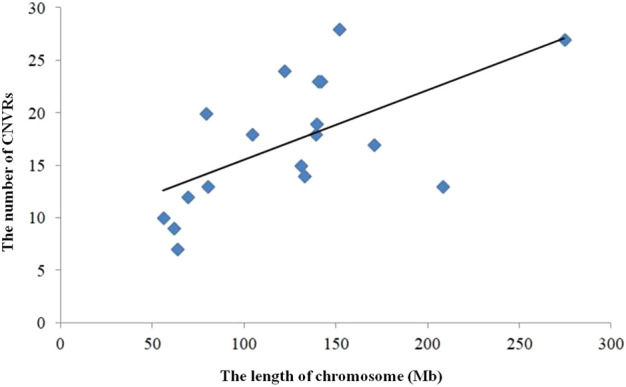
Correlation CNVR numbers and chromosome length.

The numbers of CNVRs in each autosome are presented in Table 1, and the location and characteristics of all CNVRs are displayed in Figure 2. Using regression analysis, we found a significant positive linear relationship between the chromosome sequence length and the number of CNVR located on that (*p* = 5.13E-4) (Figure 3). Longer chromosomes had higher numbers of CNVRs located on that.

These CNVRs were unevenly distributed across the whole genome. Certain chromosomal regions had a relatively high density of CNVs regions, such as each end of SSC1 and SSC2. We found a positive correlation between the number of CNVs and gene density; 77.82% of the total number of CNV regions were located on protein-coding genes, which were significantly more than non-CNV regions (*p* = 1.65E-4).

In the genomes of many mammals, SD is a necessary condition and catalyst for CNVs formation. In this study, we found that 21.44% of the CNVR sequences (25.63/119.49 Mb) directly overlapped with porcine SD regions, as obtained from the results of (Feng et al., 2017). Using Chi-squared test, we established significant enrichment of CNVRs in the SD region (*p* = 3.67E-9). It is noteworthy that CNVs are known to co-occur with SDs, and SDs are more abundant in some locations of the porcine genome. Therefore, based on our findings, we suggest that porcine CNVRs are not uniformly distributed in the genome.

In order to verify the CNVs detected by PennCNV, we utilized the QuantiSNP method to analyze the 50 K SNP data and identify CNVs. After applying the quanlity filtering criteria, we detected 2093 CNVs, and 478 CNVRs were determined by merging overlapping CNVs across all samples. The CNVRs distribution and characteristics on genome was similar to those of PennCNV. To decrease the false disvoery rate or increase the reliability of CNVR, We analyzed the overlap between the CNVRs detected by PennCNV and QuantiSNP. For 211 CNVRs identified by PennCNV, 149 CNVRs also detected by QuantiSNP, account for 70.62%. At the same time, 263 CNVRs obtained by QuantiSNP, also identified by PennCNV, account for 55.02%. The different results might be due to the different algorithms between PennCNV and QuantiSNP.

### Annotation Analysis of Duroc CNVRs

Of the 211 CNVRs we identified, 30 CNVRs did not include genes as annotated in the ENSEMBL database. The loss CNVRs were overlapping with 1,034 (26 miRNAs), the gain ones with 60 (10 miRNAs), and those involving both types with 41 (3 miRNAs) genes. The total number of genes was 1,135, including 1,096 protein-coding genes (also called CNV-gene) (such as the olfactory receptor gene family, taste receptor gene family, *DGAT1, PPARA*), and 39 miRNA genes, also called CNV-miRNA, including MIR143, MIR335, and MIRLET7.

Of those 1096 CNV-genes, 145 were dosage-sensitive genes. Using Fisher’s exact test, we found dosage-sensitive gene (*p* = 3.50E-3) enriched in CNV genes. In addition, 602 CNV-genes (54.93%) were regulated by miRNAs, while 7,872 non-CNV-genes (58.13%) were target genes of miRNAs. Therefore, target genes encompassed more non-CNV than CNV genes (*p* = 4.18E-2).

Here, we compared the expression profiles of CNV and non-CNV genes in 27 adult Duroc pig tissue types. We found that the expression of CNV genes was significant higher than that of non-CNV genes in the prostate (*p* = 2.62E-5). Of those CNV genes, 22.99% (252) were tissue-specific (TSI > 0.9). However, 24.94% of the non-CNV genes (3,390) were tissue-specific genes, which were significantly more than CNV genes (*p* = 4.99E-2).

To evaluate the functional annotation of these CNVRs, we conducted gene ontology (GO) and KEGG enrichment analyses using 1096 CNV-genes. We found 34 GO terms and seven KEGG pathways that were statistically significant (*p* < 0.05) (Table 2). Of the major GO terms 14 were associated with biological processes, 14 with cellular components, and six with molecular function categories. Significant KEGG pathways included the Hippo signaling pathway (ssc04390), Wnt signaling pathway (ssc04310), taste transduction (ssc04742), and glycerolipid metabolism (ssc00561).

**TABLE 2:** GO and KEGG pathway analyses of genes in the identified CNVRs.

Category	Term	GO or KEGG name	*p* value
Biological Process	GO:0048715	Negative regulation of oligodendrocyte differentiation	0.0039
	GO:0016192	Vesicle-mediated transport	0.0059
	GO:0007596	Blood coagulation	0.0067
	GO:0010906	Regulation of glucose metabolic process	0.0159
	GO:0051897	Positive regulation of protein kinase B signaling	0.0167
	GO:0007030	Golgi organization	0.0190
	GO:0006898	Receptor-mediated endocytosis	0.0199
	GO:0051726	Regulation of cell cycle	0.0249
	GO:0032456	Endocytic recycling	0.0256
	GO:0003151	Outflow tract morphogenesis	0.0314
	GO:0046488	Phosphatidylinositol metabolic process	0.0338
	GO:0006886	Intracellular protein transport	0.0387
	GO:0035914	Skeletal muscle cell differentiation	0.0408
	GO:0060412	Ventricular septum morphogenesis	0.0448
Cellular Component	GO:0005654	Nucleoplasm	2.60E-4
	GO:0035102	PRC1 complex	0.0010
	GO:0005769	Early endosome	0.0032
	GO:0030659	Cytoplasmic vesicle membrane	0.0070
	GO:0043235	Receptor complex	0.0071
	GO:0005604	Basement membrane	0.0135
	GO:0005581	Collagen trimer	0.0194
	GO:0005737	Cytoplasm	0.0209
	GO:0000813	ESCRT I complex	0.0226
	GO:0000791	Euchromatin	0.0226
	GO:0005829	Cytosol	0.0247
	GO:0016020	Membrane	0.0282
	GO:0000777	Condensed chromosome kinetochore	0.0328
	GO:0005925	Focal adhesion	0.0438
Molecular Function	GO:0030246	Carbohydrate binding	7.88E-4
	GO:0005096	Gtpase activator activity	0.0081
	GO:0008270	Zinc ion binding	0.0124
	GO:0005089	Rho guanyl-nucleotide exchange factor activity	0.0237
	GO:0003727	Single-stranded RNA binding	0.0239
	GO:0005509	Calcium ion binding	0.0378
KEGG Pathway	ssc04390	Hippo signaling pathway	0.0041
	ssc04310	Wnt signaling pathway	0.0131
	ssc04142	Lysosome	0.0187
	ssc04742	Taste transduction	0.0425
	ssc04520	Adherens junction	0.0443
	ssc00561	Glycerolipid metabolism	0.0463
	ssc00051	Fructose and mannose metabolism	0.0473

### Classification and Characteristics of CNV Gene Based on Structural Relationship

According to definition from Woodwark and Bateman (2011), we identified are 862 type I, 206 type II, and 28 type III CNV genes (Table 3) in this study. On average, type I CNV-genes were shorter (43.86 kb) than type II ones (201.33 kb); type III genes were the longest (461.52 kb). Obviously, type I genes were included within CNVs, whereas type III genes contained CNVs. The Kolmogorov-Smirnov test results showed that the mean or median dN plus dS values of type I genes were higher than those of non-CNV genes (Table 3). Therefore, type I genes tend to be rapidly evolving and to have increased mutation rates. Based on the dN + dS values, we also established that type II and III genes mutated more slowly than non-CNV genes (*p* = 4.24E-3, *p* = 5.64E-3, respectively). Additionally, the median or mean dN + dS values of the genes overlapping the loss type CNVRs were higher than those of non-CNV genes (*p* = 2.2E-16). Our findings evidence that type I genes overlapping loss CNVRs were rapidly evolving. The very low dN/dS values of all CNV genes would show that strong negative selection is acting on them. Those genes were remained due to genetic drift or difficulties to remove on genome.

**TABLE 3:** The mean and median values of length of genes, dN/dS, and dN + dS for the three types of CNV genes.

Gene type	# Genes	Mean(Median) length (kb)	dN/dS Mean(Median)	K-S test (*p* value)*	dN + dS Mean (Median)	K-S test (*p* value)*
Type I	862	43.86 (20.32)	0.1540 (0.1149)	7.30E-4	0.8385 (0.6717)	2.20E-16
Type II	206	201.33 (123.19)	0.1390 (0.1077)	3.30E-2	0.5199 (0.4132)	4.24E-3
Type III	28	461.52 (371.50)	0.1056 (0.0599)	1.42E-3	0.4080 (0.3146)	5.64E-3
Non CNV	13,542	71.11 (30.39)	0.1676 (0.1268)	NA	0.5910 (0.4725)	NA

* probability values for K-S test are given for the comparison of CNV gene type and non CNV genes.

According to functional annotation, type I genes tend to be involved in the regulation of glucose metabolic processes (GO:0010906), skeletal muscle cell differentiation (GO:0035914), glycerolipid metabolism (ssc00561), Wnt signaling pathway (ssc04310), and taste transduction (ssc04742). On the other hand, type II genes were associated with vesicle-mediated transport (GO:0016192), intracellular protein transport (GO:0006886), and Hippo signaling pathway (ssc04390).

### Characteristics of the Target Genes Participating in CNV-miRNAs Regulation

A total number of 39 miRNAs (called CNV-miRNAs) were located in the Duroc CNVRs. The remaining miRNAs were referred to as non-CNV miRNAs. Using Wilcox rank-sum test, we found that the number of CNV-miRNA target genes and binding sites were significantly higher than those of non-CNV-miRNA (*p*_#TargetGene_ = 1.92E-2, *p*_#BindingSites_ = 2.43E-2), respectively. Thus, CNV-miRNAs appeared to regulate more target genes than non-CNV-miRNAs.

To characterize CNV-miRNA target genes, we classified all miRNA target genes into three groups, as described earlier (Wu et al., 2012). The first target genes group had 368 genes regulated exclusively by CNV-miRNAs. Of these, 361 target genes were regulated by one CNV-miRNA, whereas the remaining target genes were regulated by two CNV-miRNAs. The second target gene group included 38 genes regulated by a combination of non-CNV mirRNAs and more than one CNV-miRNA. The third group contained 528 target genes regulated only by non-CNV miRNAs.

To investigate the target-recognition preference of CNV-miRNAs, we used a sampling simulation strategy to identify whether the observed number of target genes for each regulatory type could be expected from random sampling. These simulations provided clues for identifying the regulatory patterns of CNV-miRNAs. We found the number of target genes regulated only by two CNV-miRNAs was significantly higher than the expected after the application of random simulations (*p* = 3.57E-2). In this study, we found seven target genes that were regulated exclusively by two CNV-miRNAs, such as *CRK* gene regulated by miR-4331 and miR9817. That is, some genes are preferentially targeted by combination of some CNV-miRNAs. Obviously, the copy number alterations of one miRNA influences that of other miRNAs if their binding sites are co-located in the same UTRs. The dosage of miRNAs should be balanced to synergistically regulate the same genes.

In this study, we performed functional enrichment analyses using three groups of target genes regulated by miRNAs. ErbB signaling pathway (ssc04012) was enriched in the first group of target genes, whereas sphingolipid signaling pathway (ssc04071), NF-kappa B signaling pathway (ssc04064), and Wnt signaling pathway (ssc04310) were significantly enriched in the third group.

## Discussion

### Characteristics of the CNVRs Distribution on Duroc Genome

Recently, accumulating evidence has indicated the widespread distribution of CNVs in the genome. Furthermore, their involvement in genetic variation, phenotypic diversity, and evolutionary adaptation has been acknowledged as a major contribution (Yim et al., 2010). At least 10% of the human genome is considered to be covered by CNVs (Redon et al., 2006; Wong et al., 2007), and speculations exist that human CNVRs may cover up to 13% of the genome sequences (Stankiewicz and Lupski, 2010). In this study, 211 CNVRs were identified, which accounted for 5.23% of the autosomal sequences. Nevertheless, this figure might be conservative, because some CNVs could not be detected, including small (<10 kb) and large CNVs, which is possibly due to the small sample size and low homology probes, and as well as to limitations of current reference genomes such as sequence gaps.

Here, the abundance of loss CNVR events was approximately 5.47-fold higher than that of gain CNVR events, which is consistent with findings obtained in previous studies on cattle (Hou et al., 2011), goat (Fontanesi et al., 2010) and sheep (Hou et al., 2015). This result might be explained by action of biological factors, as suggested by (Fadista et al., 2010). Initially, non-allelic homologous recombination (NAHR) seemed to be one of the main mechanisms responsible for CNVs formation (Zhang et al., 2009). For example, Locke et al. (2006) suggested losses were under stronger selection than gains. In this respect, (Turner et al., 2008) showed that NAHR tended to generate more loss than gain. Furthermore, our results also confirm that type I CNV-genes overlapping loss CNVRs appear to be rapidly evolving.

We observed that CNVRs tended to have a non-uniform distribution in the porcine genome and were enriched in the gene density and segmental duplications regions. In the human genome, CNVRs were found to be more frequently located in some regions in the genome and chromosomes such as the pericentromeric and the subtelomeric regions (Zarrei et al., 2015). Studies have shown that the non-uniform distribution of CNVRs may arise from nearby repetitive sequences. Moreover, human CNVs were significantly overrepresented in simple tandem repeat sequences (Lupski and Stankiewicz, 2005). In primate genome, CNVs were discovered to occur together with SDs (Kim et al., 2008). Therefore, SDs may promote CNV formation (Dumas et al., 2007; Lee et al., 2008). In addition, human CNVRs were observed to be unusually enriched in protein-coding genes. The elevated gene density of CNVs might have been caused by the retention of duplicated sequences that were of adaptive benefit (Nguyen et al., 2006).

### Comparison of Our Findings With Those of Previous Studies on Porcine CNVR

Twenty studies were focused on genome-wide identification of porcine CNVs. SNP genotyping platforms, array-based comparative genomic hybridization (aCGH), and next-generation sequencing were applied in these investigations, and a total number of 16,396 CNVRs were detected, with a total length of CNVR ranging from 9.66 to 560.30 Mb in the different studies. The CNVR distributions established in each of the genome examinations are presented in Table 4. Of the 211 CNVR detected in this research, 191 had been also previously detected in earlier studies. These results indicate that approximately 90% of the CNVRs identified here can be validated by previous investigations, whereas 10% of our findings are original, first detected herein.

**TABLE 4:** Comparison of CNVRs identified in this study with previous studies.

Study	Platform	Sample	CNVR	Total length (Mb)	Average length (kb)	Range (kb)	Gain	Loss	Both	Genomic	Concordant number
Ramayo-Caldas et al. (2010)	SNP Chip	55	49	36.97/1.51%	754.59	44.70–10,700.00	19	8	22	9.2	—
Wang et al. (2012)	SNP Chip	474	382	95.76/4.23%	250.70	5.03–2,702.70	34	296	52	10.2	53
Chen et al. (2012)	SNP Chip	1,693	565	143.03/5.84%	247.55	50.39–8,100.00	225	261	79	10.2	83
Li et al. (2012)	aCGH	12	259	16.85/0.74%	65.07	2.30–1,550.00	93	140	26	10.2	18
Wang et al. (2013a)	SNP Chip	14	63	9.98/0.36%	158.37	3.20–827.21	26	36	1	10.2	6
Wang et al. (2013b)	SNP Chip	585	249	560.30/26.22%	2,305.77	29.20–27,290.00	70	43	136	9.2	—
Wang et al. (2014b)	aCGH	12	1,344	47.79/1.70%	35.56	3.37–1,319.00	557	760	27	10.2	90
Wang et al. (2014a)	SNP Chip	302	348	150.49/6.14%	443.24	4.93–12,410.00	88	243	17	10.2	57
Schiavo et al. (2014)	SNP Chip	305	170	72.33/2.95%	425.47	25.20–1700.00	7	161	2	10.2	28
Fernández et al. (2014)	SNP Chip	223	65	9.68/0.33%	148.99	3.06–1,070.00	32	21	12	10.2	11
Jiang et al. (2014)	NGS	13	3,131	102.80/4.20%	32.80	10.00–555.10	1702	1,366	63	10.2	147
Wiedmann et al. (2015)	SNP Chip	1802	502	495.29/19.1%	986.63	0.93–31727.39	—	—	—	10.2	105
Wang et al. (2015a)	NGS	49	3,131	42.10/1.72%	13.40	1.00–88.80	745	2,364	22	10.2	142
Wang et al. (2015b)	aCGH	12	758	47.43/1.69%	62.58	7.02–2,635.29	189	472	28	10.2	44
Revay et al. (2015)	SNP Chip	38	35	36.50/1.30%	1,043.73	7.47–3,755.29	5	28	2	10.2	15
Dong et al. (2015)	SNP Chip	96	105	16.71/0.68%	159.10	0.31–2,751.85	50	45	10	10.2	12
Long et al. (2016)	SNP Chip	905	737	93.70/3.82%	126.23	0.31–2,989.80	475	25 5	7	10.2	73
Revilla et al. (2017)	NGS	32	540	9.66/0.39%	17.88	3.21–1,106.44	231	305	4	10.2	34
Stafuzza et al. (2019)	SNP Chip	3,520	425	197.00/7.01%	463.62	2.50–9,718.40	19	342	64	10.2	126
Keel et al. (2019)	NGS	240	3,538	22.90/0.94%	6.80	0.23–398.90	144	3,372	22	11.1	—

The most overlapped CNVR counts (98) were consistent with those reported by (Stafuzza et al., 2019), who detected 3,520 CNVR events based on the SNP chip data of 3,520 Duroc pigs. Additionally, some studies (Chen et al., 2012; Jiang et al., 2014; Wang et al., 2015b; Long et al., 2016; Keel et al., 2019) used SNP chip or NGS platforms to identify CNVRs in Duroc pig populations. A total number of 54, 96, 88, 46, and 75 CNVR were identified in these studies that were overlapping, respectively. These results implied that these overlapping CNVRs contained some Duroc breed genome-specific CNVRs.

Remained reports have lower proportion CNVRs overlapped with our study. The issue of low overlapping rates between different reports was also occurred in CNV studies of other studies. We deem that the following reasons could have contributed to the observed differences. First, the study populations of different breeds have various genetic backgrounds. Many previous studies have also shown the presence of breed/line-specific CNVRs in the genome. (Chen et al., 2012) surveyed CNVs in 18 diverse pig populations and discovered that only 20 CNVRs of the 565 CNVRs were available in more than nine pig populations, whereas most CNVRs (72.9%) were limited to only one pig population. Second, there are differences in the sampling methods and genetic drift events among studies on the same breed. Third, different detected platforms have been used, CGH arrays, SNP genotyping, or NGS. Finally, many structural variations in the genome might have remained undiscovered.

### Duroc CNV Genes Morphology and Functions

The varying copy number of CNV genes changes gene expression due to altered gene dosage and disruption effects by gene structural variations. If CNV is located in the coding region, it alters the protein function, whereas its location in the regulatory region changes the gene expression level. Dosage sensitivity of the included genes is the most popular hypothesis that attempts to explain pathogenic CNVs. We discovered that dosage-sensitive genes were enriched in the CNV regions in the genome of Duroc pigs. The CNV regions of the human genome may be its most dosage-sensitive regions, in which CNVs are likely to be associated with disease development (Zarrei et al., 2015). However, it is worth emphasizing that changes in gene copy number do not always lead to differences in gene expression. Many factors, such as lack of regulatory elements in duplication event, the chromatin environment, and dosage compensation, might maintain stable mRNA levels.

Structural variation and miRNA are two genetic elements which affect gene expression and regulation. Here, we predicted the potential number of miRNA targets of various genes that were located either in CNV or non-CNV regions. In the genome of a Duroc pig population, we found that miRNAs regulated less CNV-gene than non-CNV gene, but the mean number of miRNA per CNV-gene is similar to that per non-CNV gene. (Felekkis et al., 2011) and Jovelin (2015) demonstrated that the genes located in the CNV regions of the human genome were targeted by more miRNA molecules, and CNV genes had more miRNA-binding sites than non-CNV genes. Similarly to the human genome, miRNA regulates more the CNV-gene in the fruit fly genome than non-CNV gene. However, Jovelin (2015) argued that this principle was not universal. In this previous study, worm and zebrafish showed the opposite pattern and had significantly more miRNAs and target sites per non-CNV genes. Therefore, structure variations such as duplication and deletion do not necessarily lead to increased miRNA target sites for CNV-gene. The distinct results among species could result from functional differences between CNV-gene and non-CNV gene, differential abundance of CNV types, and the accuracy of CNV annotations. The evolutionary interaction between miRNAs and CNVs could have been obscured by interspecies differences.

Previous reports have addressed the impact of CNVs on the phenotypic variation of domestic animals species. For instance, (Clop et al., 2012) supposed that bridging the gap between CNV genotypes and complex phenotypes will be the next genetic challenge. In addition, (Fontanesi et al., 2011) showed that duplication of the *ASIP* (agouti-signaling protein) locus was associated with a grey coat in the Massese sheep. The majority of CNVRs identified in this study overlapped with pig QTLs. Earlier, (Paudel et al., 2015) hypothesized that copy number variations provided the means for rapid adaptation to different environments during speciation/diversification. Here, we also deem that some genes with CNVs have had a possibly prominent role in the ongoing speciation, and might have impacted certain phenotypes through gene dosage alteration or *via* a positional effect, in which the structural variant might have altered the genomic landscape of the regulatory elements modulating the expression of these genes.

According to the enrichment analysis results and the already known basic gene function, genes related to some specific biological procession were identified, such as *DGAT1, DGAT2, MOGAT2, AGPAT2, FABP1, PPARA, ANGPTL3, NPC2* gene involving fat metabolic (see Table 5). *DGAT1* and *DGAT2* participate in the regulation of energy synthesis and catabolism, and affect fat metabolism and lipid deposition in tissues. *MOGAT2* is critically involved in the uptake of dietary fat by the human small intestine. The roles of *FABP1* are related to the lipid metabolism regulation by the PPAR signaling pathway. The *NPC2* gene has important functions in the transfer of cholesterol from the human lysosome.

**TABLE 5:** Some candidate genes overlapped with CNVRs involved fatness metabolic and development.

Gene symbol	Location (Mb)	Full name	Major function of involving in fatness metabolic and development
*MIR143*	SSC2: 157.34–157.34	microRNA mir143	Promote the adipogenic differentiation. The most abundant expression in developing swine adipose tissue
*MIR335*	SSC18: 19.34–19.34	microRNA mir335	Participate in the metabolism of glucose and lipid
*MIR378*	SSC2: 157.64–157.64	microRNA mir378	Participate in the metabolism of glucose and lipid
*MIRLET7*	—	microRNA let7 family	The most abundant expression in developing swine adipose tissue
*DGAT1*	SSC4: 0.60–0.61	Diacylglycerol O-acyltransferase 1	Affect fat metabolism and lipid deposition in tissues, and participate in the regulation of energy synthesis and catabolism
*DGAT2*	SSC9: 11.16–11.18	Diacylglycerol O-acyltransferase 2	Affect fat metabolism and lipid deposition in tissues, and participate in the regulation of energy synthesis and catabolism
*MOGAT2*	SSC9: 11.12–11.13	Monoacylglycerol O-acyltransferase 2	Take part in some pathway related to fat digestion and absorption and metabolism
*AGPAT2*	SSC1: 313.74–313.74	1-acylglycerol-3-phosphate O-acyltransferase 2	Associate with congenital generalized lipodystrophy, or Berardinelli-Seip syndrome
*FABP1*	SSC3: 60.62–60.63	Fatty acid binding protein 1	Role include fatty acid uptake, transport, and metabolism
*PPARA*	SSC5: 0.47–0.49	Peroxisome proliferator-activated receptor alpha	A key regulator of lipid metabolism
*ANGPTL3*	SSC1: 313.74–313.74	Angiopoietin like 3	Involve in regulation of lipid and glucose metabolism. Inhibit endothelial lipase, causing increased plasma levels of HDL cholesterol and phospholipids
*NPC2*	SSC7: 103.57–103.58	NPC intracellular cholesterol transporter 2	Plays an important role in the egress of cholesterol from the lysosomal compartment

### Functions and Regulation of Duroc CNV-miRNAs

Previous studies on human CNV discovery have reported the presence of copy number variable miRNA genes (Wong et al., 2007; Lin et al., 2008). For example, (Marcinkowska et al., 2011) found that approximately 30% of genome miRNAs were located in the human CNV regions. Additionally, (Ha et al., 2009) discovered that miRNAs had an equilibrating role in genomic dosage phenomena. The results of numerous studies have clearly evidenced the feasibility of using the dysregulation of CNV-ncRNAs as a biological marker for disease screening. In this study, we detected 39 miRNA genes that overlapped with CNVRs, including some miRNAs involved in precursor adipocyte differentiation and lipid deposition, such as MIR143, MIR335, MIR378, and MIRLET7 (Table 5). An earlier study by An et al. (2016) revealed that MIR143 was promoted the adipogenic differentiation of porcine bone marrow-derived mesenchymal stem cells. In another investigation, (Li et al., 2016) evaluated differentially expressed liver miRNAs between Tibetan and Yorkshire pigs and identified differentially expressed miRNAs (MIR335 and MIR378) that participated in the glucose and lipid metabolism. It is noteworthy that (Li et al., 2011) adopted a deep sequencing approach to determine the identity and abundance of miRNAs in swine adipose tissue development and found that MIR143 and MIRLET7 were the miRNAs with the highest expression.

Our present analysis results indicate that in the porcine genome CNV-miRNAs tend to target a higher number of genes than non-CNV-miRNAs with a pattern similar to that in the human genome, earlier established by (Wu et al., 2012). These scientists also found that this regulation model might play important roles in the prevention of CNV-miRNA purification. From an evolutionary viewpoint, certain CNV-miRNAs seem to have beneficial effects on biological processes in organisms. Our further analysis revealed that genes targeted by CNV-miRNAs participate in a wide range of biological responses to environmental factors. Obviously, CNV-miRNAs provide a possibility of increasing regulatory complexity using a strategy that increases the number target genes.

## Conclusion

In this study, we identified 211 CNVRs and constructed a CNVR map for the Duroc pig population. These CNVRs were non-randomly distributed in the Duroc genome and were significantly enriched in the segmental duplication and gene density regions. These CNVRs overlapped with 1,096 protein-coding genes (CNV-genes), 39 miRNA (CNV-miRNAs). These CNV-genes were enriched in dosage sensitivity expressed genes. Especially, the genes contained entirely within the loss CNVRs appeared to be rapidly evolving. CNV-miRNAs tended to target more genes, and a combination of two CNV-miRNAs was found to preferentially synergistically regulate the same genes. Nevertheless, further molecular experiments and independent large studies are needed to validate our findings.

## Data Availability

The variation data reported in this article have been deposited in the Genome Variation Map (GVM) in Big Data Center, Beijing Institute of Genomics (BIG), and Chinese Academy of Sciences, under accession numbers GVM000279 at http://bigd.big.ac.cn/gvm/getProjectDetail?project=GVM000279. The Bioproject accession number is PRJCA006769.
